# Placental pathology after resolved antepartum sepsis: Evidence of persistent vascular and inflammatory injury

**DOI:** 10.1016/j.placenta.2026.06.004

**Published:** 2026-06-15

**Authors:** Christine A. Blauvelt, Stephanie M. Tsoi, Kiana C. Nguyen, Sirirak Buarpung, Megan Chidboy, Nida Ozarslan, Adrienne Epstein, Michelle E. Roh, Cynthia A. Gasper, Stephanie L. Gaw

**Affiliations:** a Division of Maternal-Fetal Medicine, Department of Obstetrics, Gynecology, and Reproductive Sciences, University of California San Francisco, San Francisco, CA, USA; b Division of Critical Care, Department of Pediatrics, University of California San Francisco, San Francisco, CA, USA; c Department of Anesthesiology, Perioperative and Pain Medicine, Stanford University, Palo Alto, CA, USA; d Department of Obstetrics, Gynecology, and Reproductive Sciences, University of California San Francisco, San Francisco, CA, USA; e Department of Epidemiology and Biostatistics, University of California San Francisco, San Francisco, CA, USA; f Department of Obstetrics and Gynecology, Oregon Health and Science University, Portland, OR, USA; g Department of Pathology, University of California San Francisco, San Francisco, CA, USA

**Keywords:** Sepsis, Pregnancy, Placenta, Vascular malperfusion, Chorioamnionitis, Meconium

## Abstract

**Background:**

Sepsis during pregnancy is associated with increased risks of fetal growth restriction, preeclampsia, and placental abruption even after maternal recovery. Characterizing placental pathology after antepartum sepsis may clarify mechanisms linking infection to subsequent pregnancy complications.

**Methods:**

We performed placental histopathologic analysis on individuals delivering at a single institution. Cases had resolved antepartum sepsis (defined as clinical recovery ≥7 days before delivery), and controls were individuals without an antepartum infection hospitalization. Exclusion criteria included multifetal gestation, fetal anomalies, TORCH infections, and delivery <34 weeks’ gestation (chosen to minimize gestational age-dependent histologic variability). All placentas were reviewed by a subspecialty placental pathologist and lesions characterized according to the Amsterdam criteria. Clinical characteristics and histopathologic findings were compared using Fisher’s exact and Wilcoxon rank-sum tests.

**Results:**

Twenty-two sepsis-exposed pregnancies and 49 controls were included. The most common infection sources were urinary tract (36.4%) and respiratory (36.4%), diagnosed at a mean (SD) gestational age of 25.3 (9.2) weeks. The sepsis group was younger (31.6 [4.6] vs 34.6 [4.7] years; p = 0.017) and had higher rates of tobacco use (13.6% vs 0%; p = 0.027) and pregestational diabetes (13.6% vs 0%; p = 0.027). Sepsis-exposed placentas had significantly higher rates of maternal vascular malperfusion (31.8% vs 0%; p < 0.001), placental infarction (13.6% vs 0%; p = 0.027), avascular villi (27.3% vs 4.1%; p = 0.009), histologic chorioamnionitis (50.0% vs 17.0%; p = 0.015), and meconium-staining (50.0% vs 14.3%; p = 0.003).

**Conclusions:**

Resolved antepartum sepsis is associated with increased placental vascular and inflammatory pathology, suggesting lasting placental injury after severe systemic infection that may underlie adverse pregnancy outcomes.

## 1. Introduction

Physiologic and immunologic adaptations of pregnancy may increase susceptibility to infections such as pyelonephritis and influenza and the risk of progression to sepsis [1–3]. Antepartum sepsis is associated with maternal, fetal, and neonatal death as well as severe maternal morbidity, including intensive care unit (ICU) admission, preterm delivery, cesarean delivery, and hemorrhage [4–7]. Even among those who clinically recover prior to delivery, a history of antepartum sepsis is associated with increased risk of fetal growth restriction, preeclampsia, and placental abruption, suggesting that the dysregulated host response to infection that occurs during sepsis may disrupt placental development and function [8,9]. The mechanisms underlying placental dysfunction following resolved antepartum sepsis remain incompletely understood.

Histologic examination of the placenta may offer key insights into the pathophysiologic mechanisms responsible for this phenomenon. Early disturbances in placental development may produce characteristic maternal vascular malperfusion (MVM) lesions, particularly when insults occur during early implantation and spiral artery remodeling from 6 to 20 weeks’ gestation [10]. Inadequate uteroplacental perfusion can cause hypoxic-ischemic injury and oxidative damage, manifesting as decreased placental weight, placental infarcts, or retroplacental hemorrhage [10]. Disruption of the fetal vascular supply – through thrombosis, mechanical compression, or vessel wall damage – may produce fetal vascular malperfusion (FVM) lesions [10]. Placental histopathology can also reveal acute and chronic inflammatory responses, including chorioamnionitis, funisitis, deciduitis, villitis, and meconium staining [10]. MVM, FVM, and inflammatory placental lesions have been linked with adverse birth outcomes such as preterm birth, fetal growth restriction, stillbirth, preeclampsia, and abnormal infant neurodevelopment [10–12].

We conducted a study of individuals who experienced antepartum sepsis and recovered prior to delivery (hereafter referred to as ‘resolved antepartum sepsis’), compared with controls who did not have an antepartum infection hospitalization. The primary objective of this study was to evaluate differences in placental histopathologic findings between pregnancies with resolved antepartum sepsis and controls. We hypothesized that sepsis-exposed pregnancies would demonstrate higher rates of pathologic placental lesions.

## 2. Material and methods

This study was approved by the University of California, San FranciscoInstitutional Review Board. For sepsis cases, this study was approved with a waiver of informed consent because of the retrospective design and use of deidentified data (IRB #18-24331). Controls provided written informed consent for sample collection and review of medical records (IRB #21-33576, #20-32077). The Strengthening the Reporting of Observational Studies in Epidemiology (STROBE) reporting guideline was used when preparing the manuscript [13].

### 2.1. Study population

We conducted a study of individuals delivering at a single tertiary care center. Cases included pregnancies complicated by resolved antepartum sepsis who delivered between August 2012 and August 2018 and had placental pathology available to review. Potential cases were identified using International Classification of Diseases, Ninth Revision, Clinical Modification (ICD-9-CM) and International Statistical Classification of Diseases, Tenth Revision, Clinical Modification (ICD-10-CM) codes for infection-related hospitalizations within 10 months before delivery (Table S1 in the Supplementary Appendix). Two authors (C.A. B. and K.C.N.) reviewed electronic medical records to confirm cases of resolved antepartum sepsis. Sepsis was defined as “life-threatening organ dysfunction caused by a dysregulated host response to infection,” consistent with The Third Internal Consensus Definitions for Sepsis and Septic Shock Task Force [14]. The antepartum period was defined as the time of positive pregnancy test until onset of labor or rupture of membranes. Resolved antepartum sepsis was defined as the absence of clinical infection for at least 7 days prior to delivery.

Controls included individuals who delivered between June 2020 and April 2025 with no history of severe antepartum infection requiring hospitalization. Controls were not excluded for history of minor infections such as uncomplicated urinary tract infections or respiratory tract infections managed in the ambulatory setting. Controls were enrolled prospectively under a separate research protocol at the same institution, designed to capture a study population with maternal comorbidities reflective of the broader population delivering at our center.

Exclusion criteria for both cases and controls included pregnancies complicated by fetal structural or genetic anomalies, multifetal gestations, TORCH infections (toxoplasmosis, syphilis, varicella, parvovirus B19, Zika virus, rubella, cytomegalovirus, or herpes simplex virus), or delivery before 34 weeks’ gestation. This gestational age cutoff was chosen to reduce variability from gestational age-dependent histologic changes, as placental structure and lesions differ substantially between early preterm and late preterm/term deliveries [15–17]. Restricting the study group to ≥34 weeks’ gestation allowed for more valid comparisons between cases and controls.

### 2.2. Variables and outcome measures

Patient characteristics obtained from medical records included: age, parity, pregestational body mass index (BMI), pregestational diabetes mellitus, gestational diabetes, chronic hypertension, hypertensive disorders of pregnancy, tobacco use during pregnancy, drug use during pregnancy, and fetal growth restriction. The following obstetric outcomes were extracted from the medical records: gestational age at delivery, mode of delivery, postpartum hemorrhage, placental abruption, clinical chorioamnionitis [18], neonatal birthweight, and neonatal birthweight percentile (adjusted for gestational age and neonatal sex) [19]. Definitions are provided in Table S2 in the Supplementary Appendix.

For sepsis cases, characteristics associated with the sepsis hospitalization were also assessed including infection type, primary organ system affected, gestational age at sepsis diagnosis, and maternal ICU admission.

### 2.3. Histopathologic review

Full thickness sections from the chorionic plate to the basal plate were fixed in 10% neutral buffered formalin, paraffin-embedded, sectioned at 3 to 5 μm, and stained with hematoxylin and eosin using standard protocols. All histopathology slides from sepsis cases and controls were reviewed by a subspecialty placental pathologist (C.G.) for the purposes of this study, and placental lesions were classified according to the Amsterdam Placental Workshop Group Consensus Statement [20]. Slides were examined under light microscopy at 4x to 40× magnification. Findings of interest included: placental weight, placental weight percentile (adjusted for gestational age and neonatal sex) [21], fetal-placental weight ratio (adjusted for gestational age, parity, and neonatal sex) [22], maternal vascular malperfusion (MVM), placental infarction, accelerated villous maturation, retroplacental hemorrhage, distal villous hypoplasia, decidual arteriopathy, fetal vascular malperfusion (FVM), thrombosis, avascular villi, villous stromal-vascular karyorrhexis, vascular intramuscular fibrin deposition, stem vessel obliteration, vascular ectasia, deciduitis, chronic villitis, histopathologic chorioamnionitis, and meconium staining of the fetal membranes and chorionic plate. MVM-associated lesions evaluated in this study included accelerated villous maturation, distal villous hypoplasia, placental infarction, decidual arteriopathy, placental weight less than the 10th percentile for gestational age, and retroplacental hemorrhage. MVM was defined as the presence of ≥2 pathologic findings consistent with maternal vascular malperfusion.

### 2.4. Statistical analysis

Baseline characteristics, clinical outcomes, and placental histopathologic findings were compared between cases and controls using Fisher’s exact test for categorical variables and the Wilcoxon rank-sum test for continuous variables. Statistical analyses were performed in R (version 4.5.1; R Foundation for Statistical Computing). All statistical tests were two-sided, with statistical significance set at p < 0.05.

## 3. Results

A total of 22 individuals with resolved antepartum sepsis and 49 controls met inclusion criteria and were included in the final analysis. Baseline demographic and clinical characteristics are shown in Table 1. Individuals in the sepsis group were younger than controls (mean [SD] age, 31.6 [4.6] vs 34.6 [4.7] years; p = 0.017) and had higher rates of tobacco use (13.6% vs 0%; p = 0.027) and pregestational diabetes (13.6% vs 0%; p = 0.027). Other comorbidities, including obesity, chronic hypertension, and drug use, did not differ significantly between cases and controls. There was no significant difference in gestational age at delivery (38.6 vs 38.9 weeks; p = 0.164). Rates of antenatal and obstetric complications were also similar between the groups, including hypertensive disorders of pregnancy, gestational diabetes, fetal growth restriction, preterm delivery, cesarean delivery, postpartum hemorrhage, placental abruption, and clinical chorioamnionitis. Median birthweight was lower in the sepsis group than in controls (3000 vs 3230 g), but this difference did not reach statistical significance (p = 0.056) and birthweight percentiles were similar between the groups.

Among individuals with sepsis, the most common infection types were urinary tract (36.4%), respiratory (36.4%), and gastrointestinal or hepatobiliary infections (18.2%) (Table 2). The mean (SD) gestational age at the time of sepsis diagnosis was 25.3 (9.2) weeks. Three individuals (13.6%) required ICU admission.

Placental histopathologic findings are presented in Table 3 and Fig. 1, with representative lesions shown in Fig. 2. Compared to controls, sepsis cases were more likely to have MVM (31.8% vs. 0%; p < 0.001), placental infarctions (13.6% vs. 0%; p = 0.027), and avascular villi (27.3% vs 4.1%; p = 0.009). Syncytial knots were present in 71.4% of those with MVM and were absent in all control placentas. Sepsis cases were more likely to have FVM including 9.1% with high-grade FVM, though this difference did not reach statistical significance. Other MVM- and FVM-related findings—including decidual arteriopathy, accelerated villous maturation, thrombosis, perivillous fibrin deposition, and vascular ectasia—were more common in the sepsis group but were not statistically significant. Inflammatory lesions were more frequently observed among individuals with sepsis. Histopathologic chorioamnionitis was present in 50.0% of the sepsis group compared to 17.0% of controls (p = 0.015), and meconium staining of the fetal membranes and chorionic plate was observed in 50.0% vs 14.3%, respectively (p = 0.003).

Placental weight was lower among sepsis cases compared with controls (median 443 vs 557 g, p = 0.009) (Table 3). The distribution of placental weight percentiles did not differ between groups (p = 0.204) (Table 3). The fetal-placental weight ratio was higher in cases than controls (median [IQR]: 6.8 [6.1–8.2] vs 6.2 [5.2–7.7], p =0.062) (Table 3 and Supplemental Fig. 1). The proportion with a fetal–placental weight ratio >90th percentile was higher among cases than controls (63.6% vs 38.3%, p = 0.070), whereas the proportion <10th percentile was low in both groups (0% vs 8.5%, p = 0.299).

## 4. Discussion

Pregnancies complicated by resolved antepartum sepsis demonstrated significantly higher rates of placental vascular and inflammatory lesions compared with controls. Maternal vascular malperfusion was observed in nearly one-third of sepsis-exposed placentas and was absent in controls. Fetal vascular malperfusion was more common in the sepsis group as well, driven largely by increased presence of avascular villi. Inflammatory lesions were enriched in the sepsis group, with histologic chorioamnionitis and meconium staining each present in half of sepsis-exposed placentas. Our findings indicate that placentas from pregnancies affected by sepsis demonstrate persistent vascular and inflammatory abnormalities even after clinical recovery, providing potential pathologic correlates to the adverse pregnancy outcomes associated with antepartum sepsis [8].

### 4.1. Vascular lesions

The frequency of placental vascular malperfusion in our study population with resolved antepartum sepsis is higher than what has been previously reported in healthy pregnancies. In a study by Romero et al. evaluating 944 placentas from uncomplicated term deliveries, high-burden MVM (≥2 lesions)—which was the definition used in our study—was observed in only 7.4% of cases [23]. Similarly, Pathak et al. reported chronic placental underperfusion in 7.7% of normal pregnancies [24]. A prospective cohort study of low-risk nulliparous women demonstrated an 8.4% overall incidence of MVM lesions, with higher rates of preeclampsia and small for gestational age (SGA) infants among those with MVM placental pathology [11]. Another study of all placentas submitted for evaluation at a single institution reported MVM lesions in 15.3% and FVM lesions in 9.9% of placentas [25]. The rates of MVM and FVM were much higher in our study of sepsis-exposed placentas (31.8% and 27.3%, respectively). Additionally, our study demonstrated a trend towards higher risk of hypertensive disorders of pregnancy (27.3% in cases vs 18.4% in controls) and fetal growth restriction (18.2% in cases vs 6.1% in controls), consistent with the pathophysiologic link between vascular malperfusion lesions and clinically-significant placental dysfunction.

The pathogenesis of vascular malperfusion lesions in the setting of resolved antepartum sepsis may reflect immune dysregulation triggered by maternal infection. Successful pregnancy requires a balance between anti-pathogen responses and immune tolerance towards fetal antigens. Infections may disrupt the normal balance between pro-inflammatory and anti-inflammatory mediators in the placenta, leading to disrupted vasculogenesis and angiogenesis and manifesting as placental vascular malperfusion lesions. Dysregulation of cytokines such as interferon (IFN)-γ and tumor necrosis factor (TNF) and angiogenic factors such as vascular endothelial growth factor (VEGF), placental growth factor (PlGF), soluble fms-like tyrosine kinase-1 (sFlt-1), angiopoietin-1 (Ang-1), angiopoietin-2 (Ang-2), and soluble endoglin have been described in the setting of perinatal infections [26]. Histopathologic studies of pregnancy-associated malaria, syphilis, listeria, Zika virus, and SARS-CoV-2 infections have demonstrated abnormal placental architecture including defective spiral artery remodeling and villous malformations [10,27–32]. Notably, inflammatory remodeling can occur even in the absence of direct placental infection. Animal models of influenza have demonstrated altered placental architecture and inflammatory profiles, despite absence of viral RNA in the placental or fetal tissues [33,34]. Our findings extend this literature, demonstrating that sepsis – even when resolved weeks before delivery – leaves a durable imprint on placental structure.

### 4.2. Inflammatory lesions

Inflammatory lesions were also enriched among sepsis-exposed placentas. Histologic chorioamnionitis was observed in half of sepsis pregnancies, compared with less than one in five controls. Interestingly, clinical chorioamnionitis was more common in the control group (12.2% vs 4.5%), although this did not reach statistical significance. Clinical chorioamnionitis is typically diagnosed during labor, often in the setting of prolonged rupture of membranes with an ascending intraamniotic infection [18]. By contrast, histologic chorioamnionitis – defined by neutrophilic infiltration of the chorionic plate and fetal membranes – may represent a subclinical infectious or even sterile inflammatory process. In the context of resolved antepartum sepsis, histologic chorioamnionitis could represent persistent immune activation within the placenta that continues after maternal recovery from infection [35]. This has potential clinical implications as maternal immune activation during pregnancy may lead to aberrant immune programming in utero, ultimately contributing to abnormal development of the fetal brain, lungs, and other critical structures [36–38].

Meconium staining of the fetal membranes and chorionic plate was also notably present in 50% of sepsis cases, compared with only 14% of controls. Meconium passage may be triggered by hypoxia, intraamniotic infection and inflammation, or physiologic gastrointestinal maturation [39,40]. Chronic meconium-associated histologic changes – such as meconium-laden macrophages – may reflect fetal stress preceding labor and has been associated with placental hypoplasia and chronic fetal compromise, as compared with meconium-stained amniotic fluid which more often reflects acute intrapartum fetal distress [40,41]. Beyond serving as a marker of fetal stress, meconium is biologically active, containing bile acids and digestive enzymes that can induce vasoconstriction, endothelial damage, and even myonecrosis of umbilical and placental vessels [42,43]. Histopathologic studies demonstrate associations between meconium-related vascular injury and adverse perinatal outcomes [42,43]. In the setting of resolved antepartum sepsis, meconium staining may therefore represent both a marker of intrauterine inflammation and a mediator of placental vascular injury, linking systemic maternal infection to downstream adverse obstetric outcomes.

### 4.3. Clinical implications

Our findings demonstrate that antepartum sepsis leaves a lasting placental imprint characterized by vascular malperfusion and inflammatory lesions, providing pathologic correlates for the increased risk of adverse obstetric outcomes after resolved antepartum sepsis such as SGA and hypertensive disorders of pregnancy [8]. Even after clinical recovery from sepsis, it may be reasonable to consider closer observation for placental dysfunction – such as fetal monitoring, growth ultrasounds, blood pressure monitoring, and placental histopathology evaluation after delivery – although further studies are needed to determine whether such strategies improve outcomes.

Future research is needed to understand how the maternal, placental, and fetal immune systems respond to sepsis, and the pathophysiologic mechanisms of abnormal placental lesions after severe antepartum infections. This work may help guide novel interventions to prevent downstream sequelae of maternal infections and improve maternal and fetal outcomes.

## 5. Strengths and limitations

Our study has important limitations to consider. This was a retrospective, single institution study, with a sample size constrained by the low incidence of antepartum sepsis. Nonetheless, this study represents one of the largest series to date evaluating placental pathology following antepartum sepsis, a rare event. Because of the small sample size, we did not perform regression modeling to estimate adjusted effect sizes, and our analyses are primarily descriptive. Additionally, our study may have been underpowered to detect statistically significant differences in certain placental lesions. Future studies with larger cohorts will be important to validate and extend our findings. Another important limitation is that sepsis occurred at varying time points during pregnancy, whereas all placentas were collected at delivery. As a result, the findings represent a single time point and do not allow for temporal correlation between the timing of sepsis and the development of specific placental lesions. Finally, to reduce variability from gestational age-dependent histologic changes, we excluded deliveries <34 weeks’ gestation; however, this may have biased the study population toward less severe presentations of antepartum sepsis.

Strengths of our study include the use of cases and controls and standardized review of histopathologic specimens by a subspecialty placental pathologist with classifications according to the Amsterdam Placental Workshop Group Consensus. Additionally, our controls were not restricted to “healthy” pregnancies and included individuals with comorbidities known to affect placental function – such as obesity and chronic hypertension – at similar rates as the sepsis group. Another strength is the generalizability of the study population: most patients with sepsis were not critically ill, with only 14% requiring ICU admission, making the findings more representative of the broader obstetric population hospitalized for antepartum infections rather than limited to the most severe cases. Finally, by focusing on common infections such as urinary tract and respiratory infections, this study expands beyond the existing literature that has largely concentrated on TORCH infections.

## 6. Conclusions

Antepartum sepsis, even when clinically resolved prior to delivery, is associated with significantly increased risks of placental vascular malperfusion and inflammatory lesions. These findings suggest that severe systemic infection may induce lasting placental injury, providing potential pathologic correlates to the adverse pregnancy outcomes associated with antepartum sepsis. Future research should focus on elucidating the mechanisms of sepsis-related placental lesions and defining the long-term health consequences for exposed offspring.

## Supplementary Material

Supplemental Figure 1: Fetal weight–placenta weight relationship by sepsis exposure.

Supplementary Tables

## Figures and Tables

**Fig. 1.**
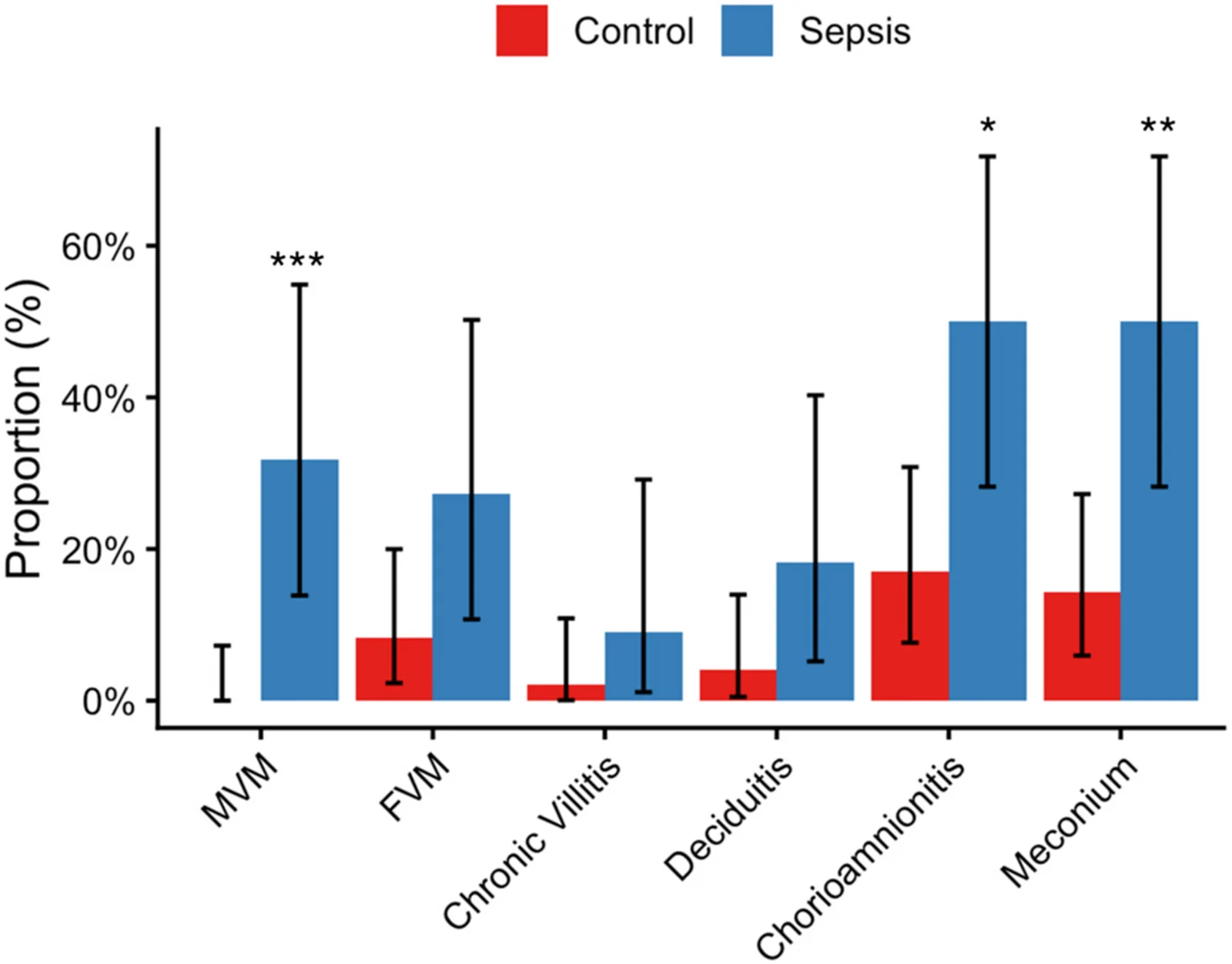
Placental vascular and inflammatory lesions in pregnancies with resolved antepartum sepsis compared with controls. Bar plots depict the proportion of placentas exhibiting each histopathologic lesion among sepsis cases (blue) and controls (red). Error bars represent 95% confidence intervals calculated using the Clopper-Pearson exact method. Significant differences are marked by asterisks (*p < 0.05; **p < 0.01; ***p < 0.001). (For interpretation of the references to colour in this figure legend, the reader is referred to the Web version of this article.)

**Fig. 2.**
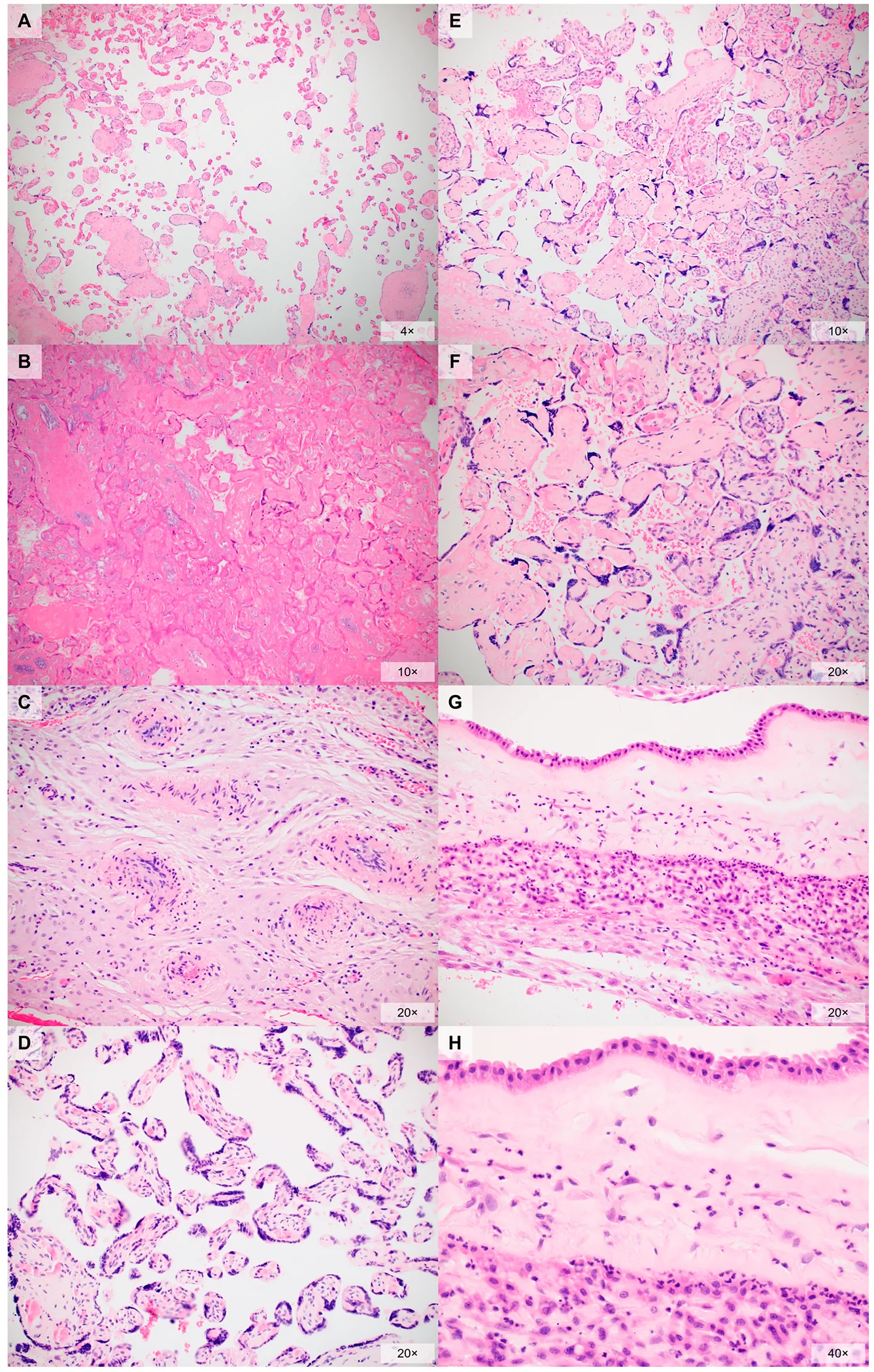
Representative placental histopathologic lesions. Hematoxylin and eosin (H&E) staining; objective magnifications are indicated. Panels A–D: Maternal vascular malperfusion, including distal villous hypoplasia (x4), placental infarction (x10), decidual arteriopathy (x20), and increased syncytial knots (x20). Panels E–F: Fetal vascular malperfusion at low (x10) and high (x20) magnification. Panels G–H: Acute chorioamnionitis at low (x20) and high (x40) magnification.

**Table 1:** Comparison of baseline characteristics and obstetric and neonatal outcomes. Data are presented as n (%), unless otherwise specified. P-values were calculated using the Fisher’s exact test for categorical variables and the Wilcoxon rank-sum test for continuous variables.

Variable	Sepsis (n = 22)	Controls (n = 49)	p-value
Maternal age – years, mean (SD)	31.6 (4.6)	34.6 (4.7)	**0.017**
Nulliparous	11 (50.0)	30 (61.2)	0.441
**Maternal comorbidities**			
Obesity (BMI ≥30)	5 (22.7)	8 (16.3)	0.524
Maternal drug use	2 (9.1)	0 (0.0)	0.093
Maternal tobacco use	3 (13.6)	0 (0.0)	**0.027**
Chronic hypertension	0 (0.0)	3 (6.1)	0.547
Pregestational diabetes	3 (13.6)	0 (0.0)	**0.027**
**Antenatal complications**			
Hypertensive disorders of pregnancy	6 (27.3)	9 (18.4)	0.530
Preeclampsia with severe features	2 (9.1)	2 (4.1)	0.583
Gestational diabetes	6 (27.3)	8 (16.3)	0.339
Fetal growth restriction	4 (18.2)	3 (6.1)	0.192
**Obstetric outcomes**			
Gestational age at delivery – weeks, mean (SD)	38.6 (1.6)	38.9 (1.1)	0.164
Preterm birth (<37 weeks)	3 (13.6)	2 (4.1)	0.169
NICU admission among preterm deliveries	0 (0.0)	1 (50.0)	0.400
Mode of delivery			0.930
Cesarean section	9 (40.9)	22 (44.9)	
Spontaneous vaginal delivery	11 (50.0)	23 (46.9)	
Operative vaginal delivery	2 (9.1)	4 (8.2)	
Postpartum hemorrhage	7 (31.8)	10 (20.4)	0.370
Placental abruption	3 (13.6)	4 (8.2)	0.669
Clinical chorioamnionitis	1 (4.5)	6 (12.2)	0.423
Neonatal birthweight – grams, median (IQR)	3000 (2813 –3316)	3230 (2965 – 3531)	0.056
Neonatal birthweight percentile – median (IQR)	34.1 (19.1 – 45.7)	38.6 (22.4 – 63.8)	0.244

BMI, body mass index (kg/m^2^); NICU, neonatal intensive care unit; IQR, interquartile range.

**Table 2:** Infection characteristics of the sepsis group.

Primary Organ System	n (%)				
Urinary tract	8 (36.4)				
Respiratory	8 (36.4)				
Gastrointestinal/Hepatobiliary	4 (18.2)				
Other	2 (9.0)				

**Table 3:** Comparison of gross and histopathologic placental findings. Placental histopathologic findings were characterized according to the Amsterdam consensus criteria. Data are presented as n (%), unless otherwise specified. P-values were calculated the Fisher exact tests.

Variable	Sepsis (n = 22)	Controls (n = 49)	p-value
**Placental weight – grams, median (IQR)**	443 (354 – 520)	557 (425 – 673)	**0.009**
**Placental weight percentile**			0.204
<10th	14 (21.5)	20 (30.8)	
10–75th	8 (9.9)	25 (30.9)	
>75th	0 (0.0)	2 (2.5)	
**Fetal-Placental Weight Ratio**			
Median (IQR)	6.8 (6.1 – 8.2)	6.2 (5.2 – 7.7)	0.062
<10th percentile	0 (0.0)	4 (8.5)	0.299
>90th percentile	14 (63.6)	18 (38.3)	0.070
**Maternal vascular malperfusion**	7 (31.8)	0 (0.0)	**<0.001**
Placental infarct	3 (13.6)	0 (0.0)	**0.027**
Accelerated villous maturation	1 (4.5)	0 (0.0)	0.310
Retroplacental hemorrhage	1 (4.5)	1 (2.0)	0.527
Distal villous hypoplasia	1 (4.5)	2 (4.1)	1.0
Decidual arteriopathy	2 (9.1)	0 (0.0)	0.093
**Fetal vascular malperfusion**			
None	16 (72.7)	44 (91.7)	0.105
Low-grade	4 (18.2)	3 (6.2)	
High-grade	2 (9.1)	1 (2.1)	
Thrombosis	2 (9.1)	0 (0.0)	0.093
Avascular villi	6 (27.3)	2 (4.1)	**0.009**
Stromal-vascular karyorrhexis	1 (4.5)	2 (4.1)	1.0
Vessel obliteration	0 (0.0)	0 (0.0)	NA
Vascular ectasia	1 (4.5)	0 (0.0)	0.310
**Perivillous fibrin deposition**	1 (4.5)	0 (0.0)	0.310
**Chronic villitis**	2 (9.1)	1 (2.0)	0.225
**Deciduitis**	4 (18.2)	2 (4.1)	0.070
**Histopathologic chorioamnionitis**			
None	11 (50.0)	39 (84.8)	
Stage 1	4 (18.2)	3 (6.4)	
≥ Stage 2	7 (31.8)	5 (10.6)	**0.015**
**Meconium staining**	11 (50.0)	7 (14.3)	**0.003**

IQR, interquartile range.

## Appendix A. Supplementary data

Supplementary data to this article can be found online at https://doi.org/10.1016/j.placenta.2026.06.004.
