# Supplementary material for: Placental pathology after resolved antepartum sepsis: Evidence of persistent vascular and inflammatory injury
Source: Placenta. Author manuscript; Available in PMC 2026 Nov 6. (PMC13741806; doi:10.1016/j.placenta.2026.06.004)
Supplement: Supplementary file 1 [file NIHMS2216855-supplement-Supplemental_Figure_1__Fetal_weight_placenta_weight_relationship_by_sepsis_exposure_.pdf]

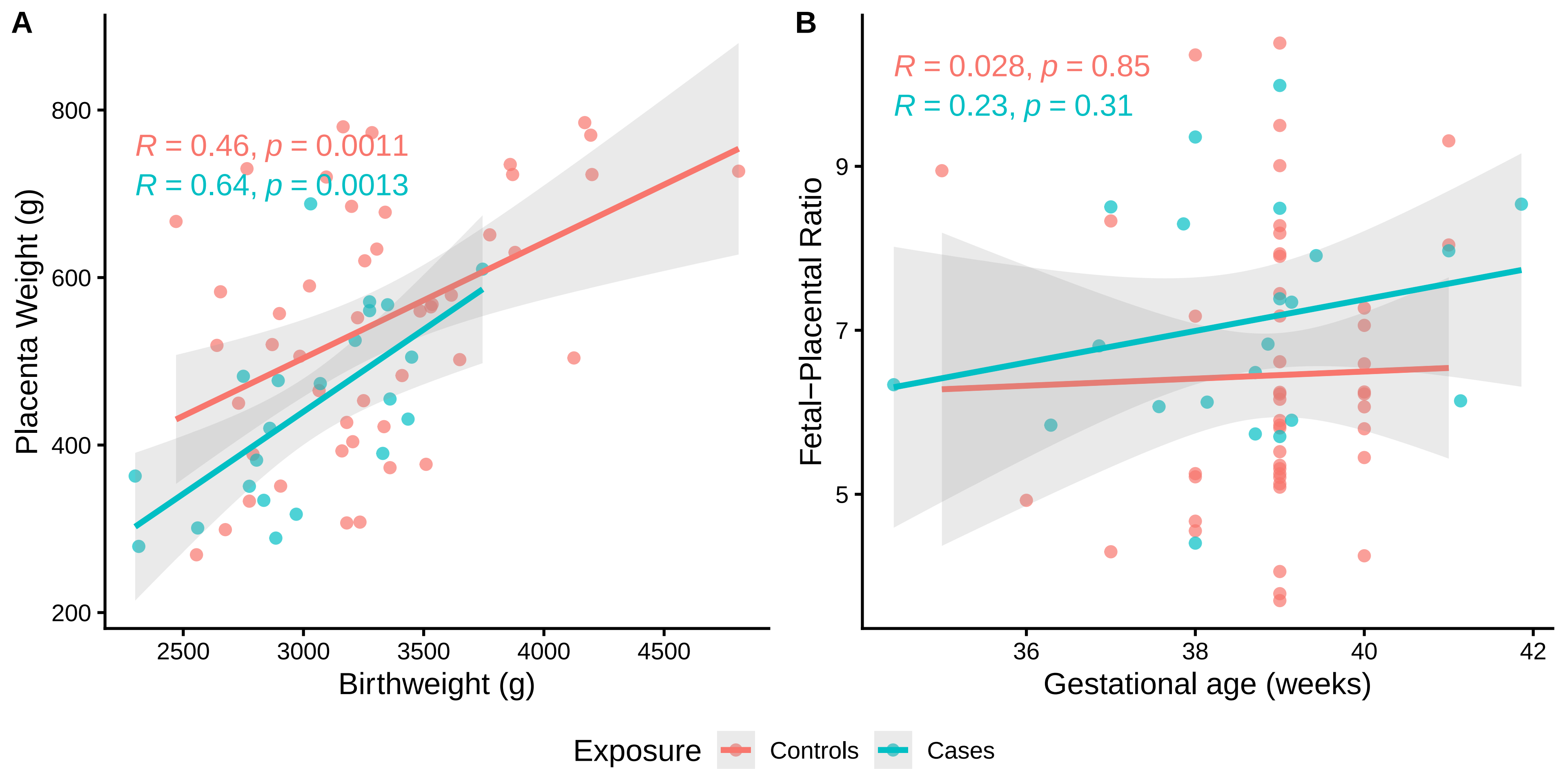
A
R = 0.46, p = 0.0011
R = 0.64, p = 0.0013
Placenta Weight (g)
800
600
400
200
2500
3000
3500
4000
4500
Birthweight (g)
B
R = 0.028, p = 0.85
R = 0.23, p = 0.31
Fetal−Placental Ratio
9
7
5
36
38
40
42
Gestational age (weeks)
Exposure
Controls
Cases
