# Supplementary material for: Placental pathology after resolved antepartum sepsis: Evidence of persistent vascular and inflammatory injury
Source: Placenta. Author manuscript; Available in PMC 2026 Nov 6. (PMC13741806; doi:10.1016/j.placenta.2026.06.004)
Supplement: Supplementary file 2 [file NIHMS2216855-supplement-Supplementary_Tables.docx]

**Supplementary Appendix**

**Table S1: *ICD-9-CM* and *ICD-10-CM* coded diagnoses for infections during pregnancy**

| *ICD-9-CM Code* | *Diagnosis* |
| --- | --- |
| 038 | Septicemia |
| 038.0 | Streptococcal septicemia |
| 038.1 | Staphylococcal septicemia |
| 038.10 | Staphylococcal septicemia, unspecified |
| 038.11 | Methicillin susceptible Staphylococcus aureus septicemia |
| 038.12 | Methicillin resistant Staphylococcus aureus septicemia |
| 038.19 | Other staphylococcal septicemia |
| 038.2 | Pneumococcal septicemia [Streptococcus pneumoniae septicemia] |
| 038.3 | Septicemia due to anaerobes |
| 038.4 | Septicemia due to other gram-negative organisms |
| 038.40 | Septicemia due to gram-negative organism, unspecified |
| 038.41 | Septicemia due to hemophilus influenzae [H. Influenzae] |
| 038.42 | Septicemia due to escherichia coli [E. Coli] |
| 038.43 | Septicemia due to pseudomonas |
| 038.44 | Septicemia due to serratia |
| 038.49 | Other septicemia due to gram-negative organisms |
| 038.8 | Other specified septicemias |
| 038.9 | Unspecified septicemia |
| 112.5 | Candida septicemia (disseminated) |
| 114 | Coccidioidomycosis |
| 114.0 | Primary coccidioidomycosis (pulmonary) |
| 487 | Influenza |
| 487.0 | Influenza with pneumonia |
| 487.1 | Influenza with other respiratory manifestations |
| 487.8 | Influenza with other manifestations |
| 590.0 | Chronic pyelonephritis |
| 590.1 | Acute pyelonephritis |
| 590.8 | Pyelonephritis NOS |
| 646.6 | Urinary tract infection complicating pregnancy |
| 646.8 | Other specified complications of pregnancy |
| 647.6 | HIV complicating pregnancy |
| 648.9 | Other current conditions complicating pregnancy childbirth or the puerperium |
| 658.4 | Infection of amniotic cavity |
| 670.2 | Puerperal sepsis |
| 760.1 | Pyelonephritis in pregnancy affecting fetus or newborn |
| 780.61 | Fever presenting with conditions classified elsewhere |
| 785.52 | Septic shock |
| 995.9 | Systemic inflammatory response syndrome (SIRS) |
| 995.91 | Sepsis |

| *ICD-10-CM Code* | *Diagnosis* |
| --- | --- |
| A32.7 | Listerial sepsis |
| A40.3 | Sepsis due to Streptococcus pneumoniae |
| A40.9 | Streptococcal sepsis, unspecified |
| A41.01 | Sepsis due to Methicillin susceptible Staphylococcus aureus |
| A41.02 | Sepsis due to Methicillin resistant Staphylococcus aureus |
| A41.1 | Sepsis due to other specified staphylococcus |
| A41.2 | Sepsis due to unspecified staphylococcus |
| A41.3 | Sepsis due to Hemophilus influenzae |
| A41.4 | Sepsis due to anaerobes |
| A41.50 | Gram-negative sepsis, unspecified |
| A41.51 | Sepsis due to Escherichia coli [E. coli] |
| A41.52 | Sepsis due to Pseudomonas |
| A41.53 | Sepsis due to Serratia |
| A41.59 | Other Gram-negative sepsis |
| A41.89 | Other specified sepsis |
| A41.9 | Sepsis, unspecified organism |
| B37.7 | Candidal sepsis |
| B38.0 | Acute pulmonary coccidioidomycosis |
| J10.1 | Influenza due to other identified influenza virus with other respiratory manifestations |
| J11.00 | Influenza due to unidentified influenza virus with unspecified type of pneumonia |
| J11.1 | Influenza due to unidentified influenza virus with other respiratory manifestations |
| J11.2 | Influenza due to unidentified influenza virus with gastrointestinal manifestations |
| J11.81 | Influenza due to unidentified influenza virus with encephalopathy |
| J11.89 | Influenza due to unidentified influenza virus with other manifestations |
| J12.9 | Viral pneumonia, unspecified |
| N10 | Acute pyelonephritis |
| N15.1 | Renal and perinephric abscess |
| O23.0 | Infections of kidney in pregnancy |
| O23.3 | Infections of other parts of urinary tract in pregnancy |
| O23.4 | Unspecified infection of urinary tract in pregnancy |
| O23.5 | Infections of the genital tract in pregnancy |
| O23.51 | Infection of cervix in pregnancy |
| O23.511 | Infections of cervix in pregnancy, first trimester |
| O23.512 | Infections of cervix in pregnancy, second trimester |
| O23.513 | Infections of cervix in pregnancy, third trimester |
| O23.519 | Infections of cervix in pregnancy, unspecified trimester |
| O23.52 | Salpingo-oophoritis in pregnancy |
| O23.529 | Salpingo-oophoritis in pregnancy, unspecified trimester |
| O23.59 | Infection of other part of genital tract in pregnancy |
| O23.599 | Infection of other part of genital tract in pregnancy, unspecified trimester |
| O75.3 | Other infection during labor |
| O98 | Maternal infectious and parasitic diseases classifiable elsewhere but complicating pregnancy, childbirth and the puerperium |
| O98.43 | Viral hepatitis complicating the puerperium |
| O98.511 | Other viral diseases complicating pregnancy, first trimester |
| O98.512 | Other viral diseases complicating pregnancy, second trimester |
| O98.513 | Other viral diseases complicating pregnancy, third trimester |
| O98.52 | Other viral diseases complicating childbirth |
| O98.53 | Other viral diseases complicating the puerperium |
| O98.7 | Human immunodeficiency virus [HIV] disease complicating pregnancy, childbirth and the puerperium |
| O98.719 | Human immunodeficiency virus [HIV] disease complicating pregnancy, unspecified trimester |
| O98.72 | Human immunodeficiency virus [HIV] disease complicating childbirth |
| O98.919 | Unspecified maternal infectious and parasitic disease complicating pregnancy, unspecified trimester |
| P00.1 | Newborn affected by maternal renal and urinary tract diseases |
| R50.81 | Fever presenting with conditions classified elsewhere |
| R50.9 | Fever, unspecified |
| R65.2 | Severe sepsis |
| R65.20 | Severe sepsis without septic shock |
| R65.21 | Severe sepsis with septic shock |
| R78.81 | Bacteremia |

**Table S2: Definitions for Clinical Characteristics and Obstetric Outcomes**

| **Variable** | **Definition** |
| --- | --- |
| Pregestational BMI | Patient-reported BMI prior to pregnancy or earliest documented BMI during pregnancy |
| Pregestational diabetes mellitus | Diabetes diagnosed prior to pregnancy or hemoglobin A1C ≥6.5% prior to 14 weeks’ gestation |
| Gestational diabetes | Abnormal glucose tolerance testing after 14 weeks’ gestation |
| Chronic hypertension | Elevated blood pressure diagnosed prior to pregnancy or before 20 weeks’ gestation |
| Hypertensive disorder of pregnancy | New elevated blood pressure of 140/90mmHg or higher after 20 weeks’ gestation |
| Tobacco use | Any patient-reported tobacco use during pregnancy |
| Drug use | Any patient-reported non-prescription drug use during pregnancy |
| Fetal growth restriction | Estimated fetal weight or abdominal circumference <10% for gestational age |
| Postpartum hemorrhage | Delivery blood loss greater than or equal to 1000mL |
| Placental abruption | Clinical diagnosis based on vaginal bleeding, uterine findings, and/or gross placental findings with or without sonographic or pathologic confirmation. |
| Clinical chorioamnionitis | Intrapartum fever and maternal tachycardia, leukocytosis >15,000 per microliter, fetal tachycardia, purulent or foul-smelling amniotic fluid, or uterine tenderness. |
